# The Role of Heat-Induced Stress Granules in the Blood–Testis Barrier of Mice

**DOI:** 10.3390/ijms25073637

**Published:** 2024-03-25

**Authors:** Zhifeng Zhao, Yuqing Cai, Xinyi Lin, Ning Liu, Yinghe Qin, Yingjie Wu

**Affiliations:** 1 College of Animal Science and Technology, China Agricultural University, Beijing 100193, China; 2 State Key Laboratory of Animal Nutrition and Feeding, China Agricultural University, Beijing 100193, China; 3 National Engineering Laboratory for Animal Breeding, China Agricultural University, Beijing 100193, China

**Keywords:** blood–testis barrier, heat stress, stress granule, UBAP2

## Abstract

Stress granules (SGs) are membraneless ribonucleoprotein (RNP)-based cellular foci formed in response to stress, facilitating cell survival by protecting against damage. Mammalian spermatogenesis should be maintained below body temperature for proper development, indicating its vulnerability to heat stress (HS). In this study, biotin tracer permeability assays showed that the inhibition of heat-induced SG assembly in the testis by 4–8 mg/kg cycloheximide significantly increased the percentage of seminiferous tubules with a damaged blood–testis barrier (BTB). Western blot results additionally revealed that the suppression of heat-induced SG assembly in Sertoli cell line, TM4 cells, by RNA inference of *G3bp1/2* aggravated the decline in the BTB-related proteins ZO-1, β-Catenin and Claudin-11, indicating that SGs could protect the BTB against damage caused by HS. The protein components that associate with SGs in Sertoli cells were isolated by sequential centrifugation and immunoprecipitation, and were identified by liquid chromatography with tandem mass spectrometry. Gene Ontology and KEGG pathway enrichment analysis revealed that their corresponding genes were mainly involved in pathways related to proteasomes, nucleotide excision repair, mismatch repair, and DNA replication. Furthermore, a new SG component, the ubiquitin associated protein 2 (UBAP2), was found to translocate to SGs upon HS in TM4 cells by immunofluorescence. Moreover, SG assembly was significantly diminished after UBAP2 knockdown by RNA inference during HS, suggesting the important role of UBAP2 in SG assembly. In addition, UBAP2 knockdown reduced the expression of ZO-1, β-Catenin and Claudin-11, which implied its potential role in the function of the BTB. Overall, our study demonstrated the role of SGs in maintaining BTB functions during HS and identified a new component implicated in SG formation in Sertoli cells. These findings not only offer novel insights into the biological functions of SGs and the molecular mechanism of low fertility in males in summer, but also potentially provide an experimental basis for male fertility therapies.

## 1. Introduction

When animals undergo temperatures that exceed their physiological tolerance and compensatory capacity, heat stress (HS) occurs [1]. The testis is a temperature-sensitive organ that needs to be maintained 2–7 °C below core body temperature to ensure normal spermatogenesis [2]. Therefore, the testis is more susceptible to HS than other tissues. There is plenty of evidence that HS results in defects in testicular development, the arrest of spermatogenesis, worsened semen quality, and infertility in males [3,4,5]. Sertoli cells serve as primary supportive cells within the seminiferous epithelium, offering vital nutrients and assistance to spermatogenic cells. The adjacent Sertoli cells near the basement membrane form a special cellular junction, the blood–testis barrier (BTB). The BTB is constituted by tight junctions, adherens junctions (also known as basal ectoplasmic specialization), gap junctions and desmosomes [6,7,8,9,10]. The function of BTB is to restrict substances, such as nutrients, vital molecules and harmful toxicants, into the apical compartment, providing a unique microenvironment for post-meiotic spermatid development. An increase in testicular temperature triggers ultrastructural damage to the BTB and the reversible disruption of the function of Sertoli cells. For example, after the heat treatment of Sertoli cells, a notable decrease occurs in both mRNA and protein levels of the tight junction-related proteins Occludin and ZO-1, leading to the disruption of the BTB structure and an increase in BTB permeability [11]. Heat-induced cell junction disruption can be rescued by androgen receptor overexpression, which acts upstream to regulate the reversible change in the BTB by rescuing the expression of ZO-1, N-Cadherin and E-Cadherin [12].

Stress granules (SGs), ribonucleoprotein (RNP) granules, manifest in cells as a response to stressors like chemical injury, oxidative stress and viral incursion [13]. SGs form from mRNAs stalled in translation initiation and are composed of multiple constituents, including translationally stalled mRNAs, translation initiation factors, RNA-binding proteins and non-RNA-binding proteins [14]. However, the molecular composition and regulatory dynamics of SGs vary with cellular context or stressor type [15]. For example, G3BP1 and G3BP2 serve core functions in facilitating SG assembly within mammalian cells when subjected to oxidative stress, while they are not required during osmotic stress [16,17]. Moreover, Gtr1, Rps1b, and Hgh1 promote SG formation during glucose starvation while repressing SG formation during heat shock in yeast [18]. And it should be noted that the way in which SG assembly affects gene expression and participates in cellular physiology remains to be determined.

A growing body of studies has revealed that persistent aberrant SG formation or mutations in nucleators contribute to neurodegenerative diseases, including amyotrophic lateral sclerosis and Alzheimer’s disease [19,20,21], while the implication and function of SGs in spermatogenesis have been seldom elucidated. Kim et al. report that DAZL acts as a specific translational regulator and localizes to SGs during HS, curbing germ cell apoptosis by sequestering signaling molecules such as RACK1 [22]. Another testis-specific protein, MAGE-B2, has been proven to enhance the cellular stress threshold and protect this highly thermosensitive germline from heat stress by suppressing G3BP1 translation and SG assembly in germ cells [23]. However, insights from these studies about SG function in the male reproductive system remain limited. Gaps remain in our understanding of SG components, regulation and function in germline cells and somatic cells (e.g., Sertoli cells) during spermatogenesis.

Thus, this study aims to investigate the implication and function of SGs in Sertoli cells by elaborating their formation, composition and effects on BTB integrity and BTB protein expression during HS. We found that inhibition of SG formation with an inhibitor or siRNAs resulted in the impairment of the BTB and a reduction in BTB-related proteins. Moreover, we found that UBAP2 promoted SG formation by maintaining the expression of the SG nucleator G3BP1 and the deficiency of UBAP2 further exacerbated the decrease in BTB-related proteins. These results firstly demonstrate the role of SGs in maintaining BTB functions and identified a new component that is essential for SG formation in Sertoli cells upon heat stress.

## 2. Results

### 2.1. Heat Stress Disturbs Structure of Seminiferous Epithelium and Integrity of BTB

**Figure 1:**
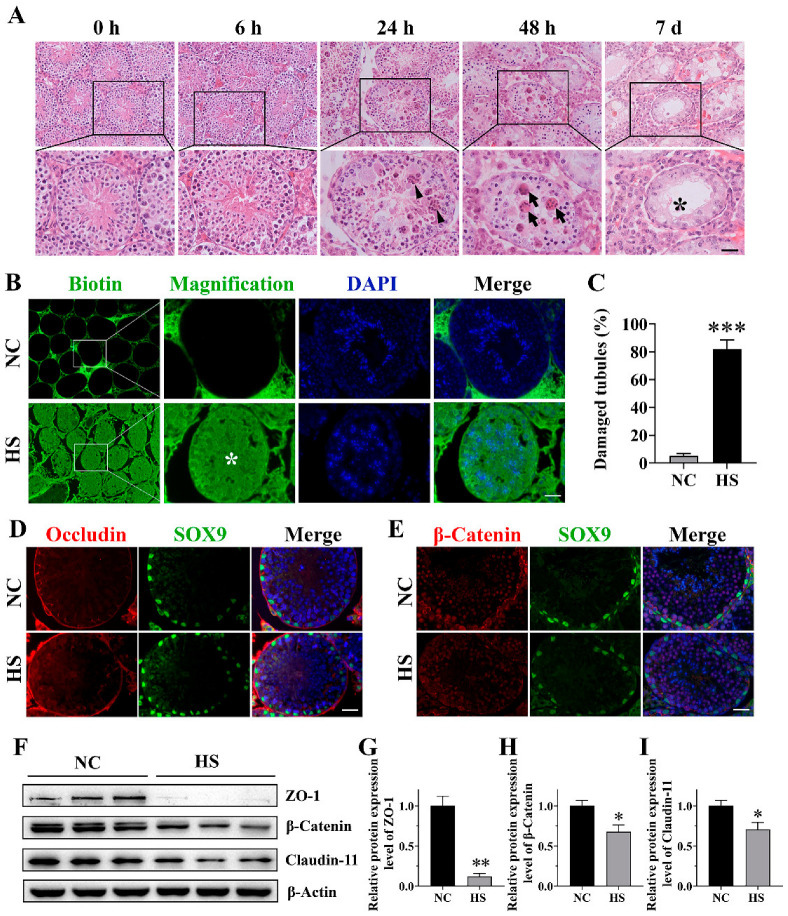
Heat stress (HS) led to disorganized structure of seminiferous epithelium and blood–testis barrier (BTB) disruption. (**A**) Histological changes in testes after different recovery time. The lower 1/3 of the mice were immersed in a water bath of 43 °C for 30 min followed by recovery for 0 h, 6 h, 24 h, 48 h and 7 d. Triangles: abnormally aggregated spermatogenic cells; arrows: multinuclear giant cells; asterisk: Sertoli-cell-only-like seminiferous tubule. This experiment was repeated 3 times. Scale bars, 50 μm. (**B**,**C**) Changes in BTB integrity in the testis. BTB integrity was assessed after 24 h recovery from HS treatment using biotin tracer permeability assay. Biotin was detected by coupling to streptavidin-FITC (green), and nuclei were labeled with DAPI (blue). Quantification of damaged tubule was performed based on the cross-section of the testis. (**D**,**E**) Localization of Occludin and β-Catenin in testes. SOX9 is used to mark the nucleus of Sertoli cells. (**F**–**I**) Immunoblots and quantitative analysis of ZO-1, β-Catenin and Claudin-11 in testis extracts between control and HS group. * = *p* ≤ 0.05, ** = *p* ≤ 0.01, *** = *p* ≤ 0.001.

In order to evaluate the effect of HS on the function of Sertoli cells, we firstly investigated the effects of HS on testicular histology and BTB integrity. As shown in Figure 1A, HS did not cause obvious histological changes in the seminiferous epithelium immediately or 6 h post-recovery. However, after 24 h of recovery from HS treatment, the seminiferous tubules were apparently disorganized, accompanied with the abnormal aggregation of spermatogenic cells. And after 48 h recovery from HS, many multinuclear giant cells were observed, and spermatogenic cells became scattered and thin, suggesting that these cells probably underwent apoptosis and the BTB may have disintegrated. After 7 d recovery, more severe degradation was detected in most of the seminiferous tubules, as indicated by the frequent occurrence of Sertoli-cell-only-like seminiferous tubules.

To evaluate the integrity of the BTB after HS, a biotin tracer was injected into the testes of 8-week-old mice. As expected, in the testes of the control mice, the tracer was restricted to the interstitium and the basal compartment of the seminiferous tubules. However, in the testes of the HS mice, the tracer was detected in the entire seminiferous epithelium and lumen in some seminiferous tubules (Figure 1B), which indicated that the BTB was disrupted. Quantification showed that the number of seminiferous tubules with a damaged BTB was significantly increased in the HS mice testes (81.77 ± 6.76%) when compared with that in the control mice testes (5.19 ± 1.55%, Figure 1C). After that, we examined the localization and expression of BTB-related proteins. After HS, Occludin (Figure 1D) and β-Catenin (Figure 1E), which should be linearly distributed in the spermatogenic epithelium adjacent to the basal compartment, were disturbed by HS. The BTB-related protein levels of Claudin-11, ZO-1 and β-Catenin were significantly (*p* < 0.05) reduced 24 h after HS when compared to the control group (Figure 1F–I). These findings prove that the integrity of the BTB is significantly disrupted by HS.

### 2.2. Heat Stress Induces SG Assembly in Sertoli Cells and Is Reversed by Cycloheximide

**Figure 2:**
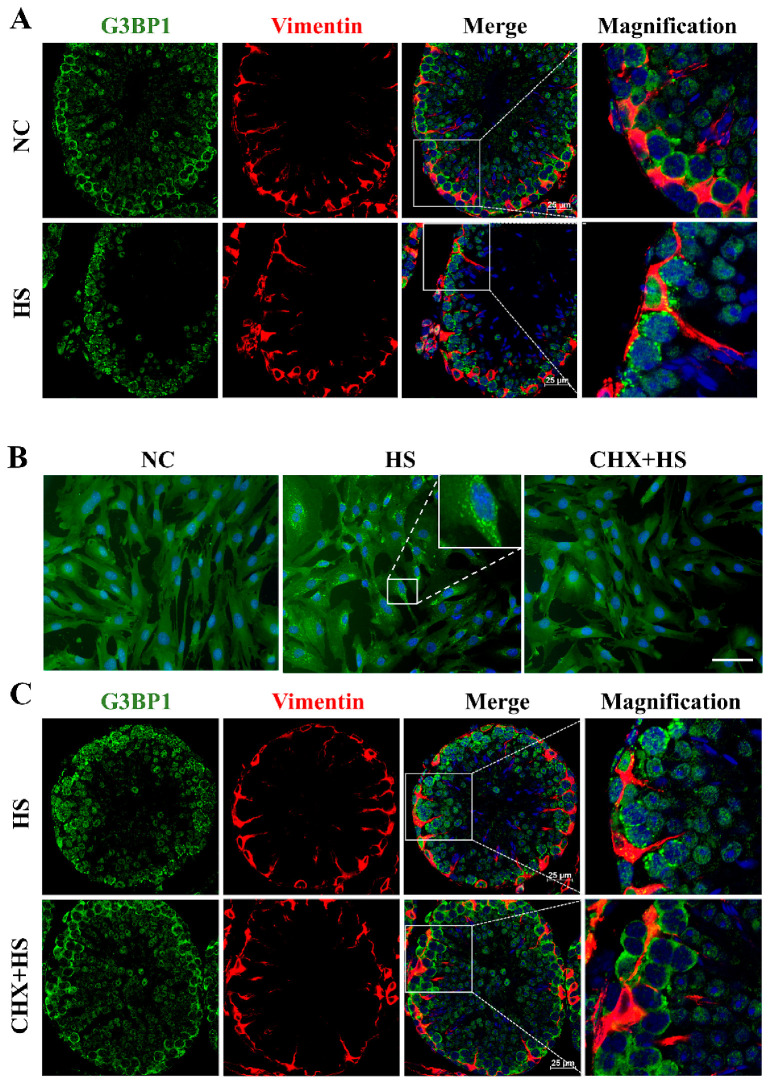
HS induced stress granule (SG) assembly in Sertoli cells. (**A**) The SG assembly in testicular cells. The testes of the mice were subjected to HS and fixed in 4% paraformaldehyde. Testicular sections were co-stained for Vimentin (red) and G3BP1 (SG markers, green). (**B**) The SG assembly in Sertoli cells in vitro. The Sertoli cells were isolated from 20-day-old mice testes and cultured in DMEM/F12. After different treatments (control, 43 °C for 30 min, 20 μg/mL CHX and 43 °C for 30 min), they were fixed and stained for G3BP1 (green). Scale bars, 25 μm. (**C**) The SG formation in testicular cells was inhibited by CHX. The mice were subjected to HS after saline or CHX (8 mg/kg, tail vein injection) injection.

Although it has been reported that HS induces SG assembly in spermatogonia and spermatocytes [22], whether HS triggers SG assembly in Sertoli cells is still unknown. G3BP1, known as the central regulator of SG assembly, is used to indicate SG formation. In the normal testes, G3BP1 was distributed homogeneously in the cytoplasm of the spermatogonia, spermatocytes and Sertoli cells (Figure 2A). When the testis was exposed to HS, G3BP1 assembled into cytoplasmic granules in the cytoplasm of the spermatogonia, spermatocytes as well as Sertoli cells. The cytoplasm of Sertoli cells was indicated by immunostaining of Vimentin (Figure 2A). In vitro, the Sertoli cells showed noticeable SG formation at 43 °C (Figure 2B). SG formation was detected in more cells after HS (91.87 ± 2.48%) than in the untreated controls (1.01 ± 1.51%), whereas the assembly of heat-induced SGs was reversed (1.05 ± 1.04%) by cycloheximide (CHX, 20 μg/mL), an inhibitor of SG formation. Notably, in vivo CHX inhibited SG production in all the cells of the testis, including spermatogenic cells and Sertoli cells (Figure 2C). Taken together, these data prove that HS triggered SG assembly in Sertoli cells.

### 2.3. The Role of SGs in Heat-Induced BTB Damage

**Figure 3:**
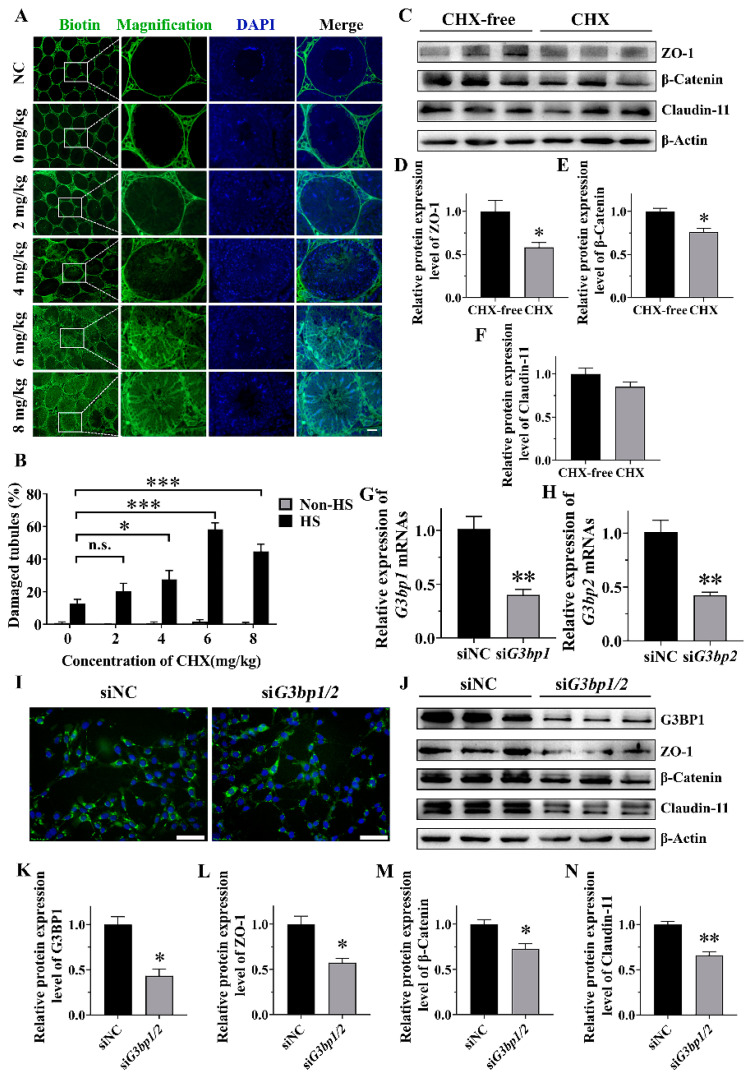
Inhibition of SG assembly aggravated BTB injury and decreased the expression of BTB-associated protein under elevated temperature. (**A**,**B**) Changes in BTB integrity in the testes. The testes of the mice were subjected to HS after the injections of CHX/saline and anesthesia. The biotin tracer permeability was used to assess the integrity of the BTB. Scale bars, 50 μm. (**C**–**F**) Immunoblots and quantitative analysis of ZO-1, β-Catenin and Claudin-11 in cultured TM4 cells after CHX treatment. TM4 cells were subjected to three consecutive repeated treatments, with each treatment consisting of heating at 43 °C for 30 min in medium with or without 20 μg/mL CHX (heat) followed by maintenance at 37 °C for 7 h in complete medium (recovery). (**G**,**H**) Knockdown efficiency of si*G3bp1* and si*G3bp2* at mRNA level in TM4 cells. (**I**) Effect of si*G3bp1* plus si*G3bp2* (si*G3bp1*/*2*) on SG assembly in TM4 cells during HS. Cells were transfected with siNC or si*G3bp1*/*2* for 48 h followed by HS at 43 °C for 30 min. Scale bars, 25 μm. (**J**–**N**) Immunoblots and quantitative analysis of G3BP1, ZO-1, β-Catenin and Claudin-11 in siNC- and si*G3bp1*/*2*-treated TM4 after HS treatment. Cells were transfected with siNC or si*G3bp1*/*2* for 24 h followed by three repeated heat recovery treatments, as above. * = *p* ≤ 0.05, ** = *p* ≤ 0.01, *** = *p* ≤ 0.001, n.s. = not significant.

Considering that HS caused both SG assembly and BTB damage, we explored further the effect of the suppression of SG assembly on the BTB by the injection of CHX before HS treatment. The results showed that the percentage of seminiferous tubules with a damaged BTB was extremely low in the untreated control and the CHX-only control, and was elevated moderately (12.68 ± 2.73%) after heat treatment (Figure 3A,B). However, when SG assembly was inhibited by 4, 6 and 8 mg/kg CHX under HS, the damage of the BTB increased significantly (Figure 3A,B). Specifically, the percentage of damaged tubules increased with dose and peaked at 6 mg/kg CHX (58.33 ± 3.90%). The Western blot results showed that the BTB-related proteins ZO-1 and β-Catenin were significantly reduced in SG-assembly-inhibited groups compared to the control under HS (Figure 3C–E). However, the expression of Claudin-11 was not affected. Nevertheless, these results suggests that the suppression of SG formation in vivo exacerbates BTB damage and decreases the expression of ZO-1 and β-Catenin.

To further confirm the importance of SG assembly in BTB regulation, the expression of nucleating proteins of SGs, G3BP1 and G3BP2, were knocked down in TM4 cells using siRNA in order to break up the formation of SGs. The RT-qPCR results verified the expression of *G3bp1* and *G3bp2* was down-regulated (Figure 3G,H) and the Western blot results further confirmed the decline of G3BP1 at the protein level (Figure 3J,K). As expected, compared with siNC, SG assembly was significantly decreased (from 82.03 ± 5.56% to 65.35 ± 7.36%) in si*G3bp1*/*2* when the cells were subjected to HS (Figure 3I and Figure S1). When SG assembly was inhibited by si*G3bp1*/*2* during HS, the protein levels of ZO-1 and β-Catenin were significantly reduced (Figure 3J,L,M); even the expression of Claudin-11 was moderately down-regulated (Figure 3J,N). These results indicate that the inhibition of heat-induced SG assembly in vitro results in the decreased expression of BTB-related proteins.

### 2.4. Identification of Heat-Induced SG Components in TM4

**Figure 4:**
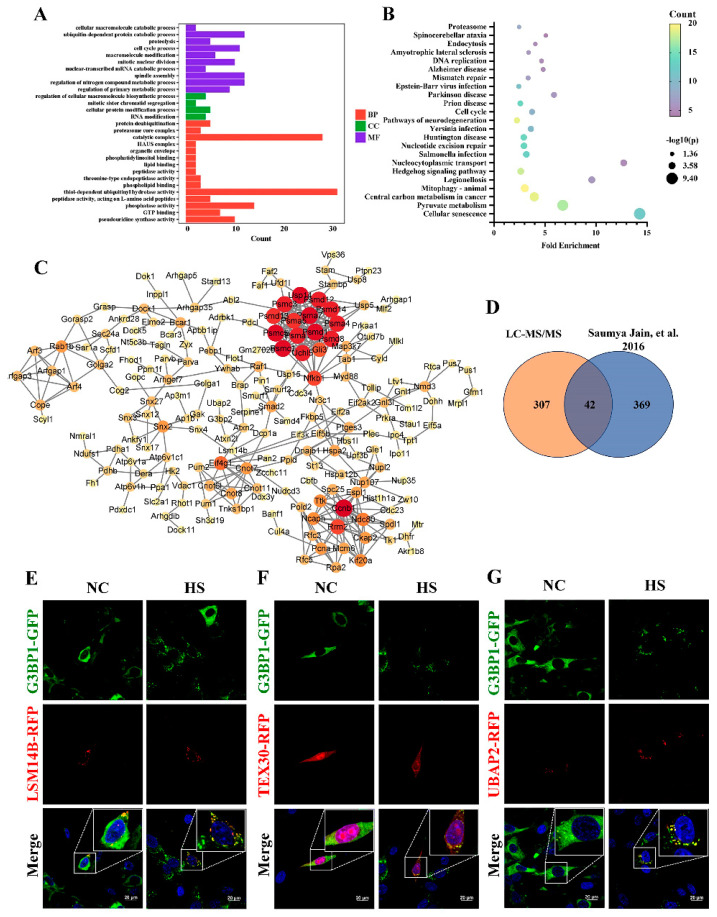
Proteome analysis revealed the SG components in TM4 cells after HS. (**A**) Gene Ontology analysis of identified proteins in TM4 SGs. SGs in TM4 cells with stable expression of G3BP1-GFP were induced at 43 °C for 30 min and isolated through sequential centrifugation. The protein components were further enriched by immunoprecipitation and identified by LC-MS/MS. BP: biological processes; CC: cellular component; MF: molecular functions. (**B**) The KEGG pathway analysis of identified proteins. (**C**) Protein–protein interactions of specific enriched proteins in SGs. Dataset was predicted by STRING 10.0 database (confidence score > 0.7). (**D**) Comparative statistics of SGs in TM4 cells and U-2 OS cells [14]. (**E**–**G**) Co-localization of G3BP1 with the candidate protein (LSM14B, UBAP2 and TEX30) in TM4 cells. Each of candidate genes was constructed into pDsRed1-C1 vectors with red fluorescent protein sequence, and then was transfected into G3BP1-GFP TM4 cells. After 48 h, cells were treated at 43 °C for 30 min and fixed.

To unravel the protein composition within the “core” of SGs induced by HS in TM4, a cell line stably expressing GFP-G3BP1 via lentiviral transduction was generated. The “core” was isolated through sequential centrifugation and immunoprecipitation, followed by liquid chromatography with tandem mass spectrometry. From proteome analysis, it was found that there were 349 proteins detected only in the HS group *(p* < 0.05) compared with the unstressed group (Supplementary Table S1). The identified proteins were significantly enriched in 29 GO terms (*p* < 0.05, Figure 4A and Supplementary Table S2), including 15 biological processes, 4 cellular components and 10 molecular functions. The enriched proteins primarily function in RNA binding activity and protein metabolism, such as nuclear-transcribed mRNA catabolic processes, RNA modification, ubiquitin-dependent protein catabolic processes and proteolysis in the GO analysis. The KEGG pathway analysis revealed 23 enriched pathways which were involved in the proteasome, DNA replication, mismatch repair, the cell cycle and so on (*p* < 0.05, Figure 4B and Supplementary Table S3).

To explore the functional characteristics and network of the enriched proteins in the SGs, the networks were constructed and visualized by Cytoscape (v3.9.1), based on STRING 10.0 (Figure 4C). The proteins associated with proteasomes, such as the PSMD family, perform important roles in the interaction network. Proteasomes cleave peptides in an ATP/ubiquitin-dependent process in a non-lysosomal pathway, suggesting a potential protein degradation pathway for SGs. Next, the statistical analysis showed that 42 proteins overlapped with NaAsO_2_-induced SGs in U-2 OS cells, in accordance with a previous study, suggesting the presence of similar components in different cell types, including classical nucleation factors such as G3BP2 and G3BP1 (Figure 4D) [14]. However, there were also some testis-specific proteins such as PTGES3 and TEX30 that play potential roles in the testis. To further validate the identified results, we randomly selected seven candidate proteins for which to construct an individual expression vector that facilitated the expression of their fusion to red fluorescent protein (RFP), and transfected each of them into TM4 cells. Our observations showed that three (UBAP2, TEX30 and LSM14B) out of the seven proteins co-localized with G3BP1-GFP during HS, thus establishing them as novel constituents of SGs (Figure 4E–G).

### 2.5. The Role of UBAP2 in the Assembly of Heat-Induced SGs and the Expression of BTB-Associated Proteins

**Figure 5:**
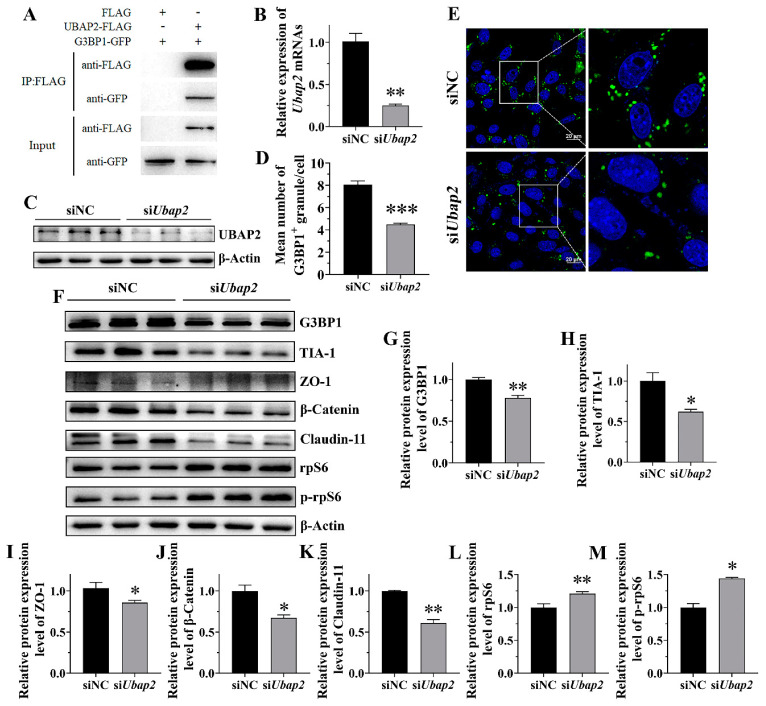
UBAP2 knockdown attenuated SG assembly and aggravated the decrease in BTB-associated protein in response to HS. (**A**) UBAP2 interacted with G3BP1 in SGs. HEK293T cells were transfected with indicated plasmids. Immunoprecipitation was performed using anti-FLAG resin 24 h after transfection. GFP-G3BP1 and FLAG-tagged proteins were detected by Western blotting with anti-GFP and anti-FLAG antibodies. (**B**,**C**) Knockdown efficiency of si*Ubap2* at mRNA level (**B**) and protein level (**C**) in TM4 cells. (**D**,**E**) Effect of si*Ubap2* on SG assembly in TM4 cells during HS. Cells were transfected with siNC or si*Ubap2* for 48 h followed by HS at 43 °C for 30 min. (**F**–**M**) Immunoblots and quantitative analysis of G3BP1, TIA-1, ZO-1, β-Catenin, Claudin-11, rpS6 and p-rpS6 after HS treatment. Cells were transfected with siNC or si*Ubap2* for 24 h followed by three repeated heat recovery treatments, as above. * = *p* ≤ 0.05, ** = *p* ≤ 0.01, *** = *p* ≤ 0.001.

Based on above results, we chose UBAP2, the ubiquitin associated protein 2, to further investigate its potential functions in SG assembly. Firstly, we confirmed its direct association with the G3BP1 protein. The Co-IP results showed that G3BP1-GFP can be pulled down by UBAP2-FLAG but not by FLAG control, indicating the interaction between UBAP2 and G3BP1 (Figure 5A). And then, the expression of UBAP2 in the TM4 cells was knocked down using siRNA (Figure 5B,C). The RT-qPCR and Western blot results validated the efficiency of the UBAP2 mRNA and protein (Figure 5B,C). Importantly, UBAP2 knockdown significantly decreased the SG number after HS treatment, as indicated by the immunostaining for G3BP1 and the quantification of G3BP1-positive granules in the cells (Figure 5D,E), while it had no effect on SG disassembly (Figure S2). In order to explore the potential mechanism of UBAP2 affecting SG assembly, we detected the expression levels of G3BP1 and TIA-1 by Western blot in siNC and si*Ubap2* TM4 cells. The results showed that the knockdown of UBAP2 reduced the expression of G3BP1 under HS (Figure 5F,G) but not under normal conditions (Figure S3), suggesting that UBAP2 may promote SG assembly through interacting with and maintaining the expression of G3BP1 during HS, while UBAP2 knockdown reduced the expression of TIA-1 under both HS (Figure 5F,H) and non-heat stress conditions (Figure S3).

To further study the roles of UBAP2 in BTB regulation, we determined the expression of several BTB-related proteins by Western blot before and after UBAP2 interference. The result showed that the protein expression of ZO-1, β-Catenin and Claudin-11 were significantly decreased (*p* < 0.05) in the si*Ubap2* group compared with the control during HS (Figure 5F,I–K). Moreover, the expression of rpS6 and p-rpS6, a crucial downstream signaling molecule of mTORC1, was significantly increased after UBAP2 knockdown (Figure 5F,L,M). These results thus support that UBAP2 is a critical regulator of the BTB under HS.

## 3. Discussion

Although many studies have identified an association between heat exposures and low fertility in males, few studies have focused on the function of SGs in spermatogenesis. It is reported that SGs can protect germ cells because the percentage of apoptotic germ cells in the testis increases significantly in the absence of DAZL and SG formation [22]. But in another study, the testis-specific protein MAGE-B2 increases cellular stress tolerance through the suppression of SG assembly by translationally inhibiting the SG nucleator G3BP [23]. Whether SGs play a positive or negative role in spermatogenesis remains difficult to determine. Our results have shown that the suppression of SG assembly aggravates BTB damage and decreases BTB protein expression, and have also emphasized the role of SGs in Sertoli cells during the maintenance of the functional BTB.

Sertoli cells, directly in contact with germ cells, provide the microenvironment and nourishment necessary for spermatogenesis. Testicular injury is mild just after experiencing acute HS, but can become more severe in the following recovery period, as shown by the appearance of multinuclear giant cells after 48 h and Sertoli-cell-only-like seminiferous tubules after 7 d in the mice in our experiments. This result agrees with a previous study suggesting that the testes experience a lasting negative effect after a single period of mild HS. The testes of mice were stressed at 42 °C for 30 min, and a histological examination revealed multinucleated giant cells, germ cell depletion and collapsed seminiferous epithelium in the testes at 24 h, 48 h and 7 d after HS, respectively. It was also suggested that TUNEL-positive cells might burst at 24 h after heat stress [24]. However, the changes in the BTB were not examined in that study. As a supplement, our study proved that HS caused the breakdown of the BTB and disrupted the expression of the BTB-related proteins Claudin-11, ZO-1 and β-Catenin in the testis.

Eukaryotic cells exhibit stereotypical responses to various stressors, aiding their survival under extreme conditions and during their subsequent recovery [25]. While SG assembly is a universal process in response to different cellular stresses [26], previous research has demonstrated HS-induced SG assembly primarily in male germ cells and early spermatocytes indicated by the staining of TIA-1 [22]. As an extension, our study proved that both Sertoli cells and germ cells form obvious SGs after treatment at 43 °C for 30 min. Since Sertoli cells are responsible for nourishing spermatogenic cells and have no fixed morphology, their boundaries are difficult to determine. It is difficult to detect whether Sertoli cells generate SGs in response to HS. We therefore used Vimentin, a cytosolic marker of Sertoli cells [27], to determine Sertoli cell boundaries and confirmed SG formation during HS.

In order to investigate the effects of SGs on the BTB integrity and BTB-related protein expression under HS, CHX was used as an inhibitor to suppress SG formation in vivo. CHX can inhibit SG assembly by interrupting mRNA–polysome interaction and is frequently used as an inhibitor of SG assembly in vitro [28,29,30]. The present results also suggest it can inhibit the formation of cellular SGs in vivo. To support this finding, we used another method to inhibit the formation of SGs in vitro: double interference of *G3bp1* and *G3bp2*. G3BP1 and G3BP2 have been identified as playing a central role within the core SG network. A study examined the importance of 36 proteins in the core SG network, using CRISPR/Cas9 to knock out one or both isoforms of their genes in the U2OS cell line. The result suggested that the cells only with the double knockout of *G3bp1* and *G3bp2* are devoid of SGs [31]. In our study, G3BP1/2 in TM4 cells was knocked down by siRNA and resulted in a reduction in the SG-positive cells from 82% to 65% during HS. Therefore, we suppressed SG assembly both in vivo and in vitro, and proved the protective function of SGs in Sertoli cells during the maintenance of the functional BTB.

For the purification of SGs, we established a TM4 cell line stably expressing G3BP1-GFP and isolated SGs according to the method described by Wheeler et al. [32,33]. LC-MS/MS identified 349 possible core proteins, as assessed with a *p* < 0.05 compared to the unstressed control. The statistical analysis showed that at least 42 of the identified proteins overlap with those found in a previous study, many of which have been identified as SG proteins [14]. Based on previous studies, ubiquitination and the proteasome play critical roles in the assembly of SGs, which are pivotal for the restoration of cellular functions following HS [34,35]. In this study, some enriched proteins related to ubiquitination and the proteasome highly expressed specifically in the testes (as shown in a public database) were found in the SGs. Meanwhile, novel components within SGs in Sertoli cells were identified, including UBAP2, TEX30 and LSM14B, which were highly expressed in the testes and had not been reported to exist within SGs previously.

SG assembly is triggered by liquid–liquid phase separation arising from interactions distributed unevenly within the protein–RNA interaction network. The molecular switch of this network, G3BP1, triggers RNA-dependent LLPS when concentrations of intracellular free RNA rise [31]. Other G3BP1-binding factors further regulate SG assembly by strengthening or weakening the core network of the SGs through positive or negative cooperativity. In this study, the knockdown of UBAP2 resulted in a significant reduction both in the quantity of SGs and in the expression of G3BP1 during HS, while it did not affect the expression of G3BP1 in normal conditions. Therefore, we proposed that UBAP2 probably acts upstream of G3BP1 to maintain SG assembly in Sertoli cells in response to HS.

The knockdown of UBAP2 also further suppressed the expression of Claudin-11, ZO-1 and β-Catenin during HS. This can be explained by the decreased expression of rpS6 and rpS6 phosphorylation. As a downstream member of the mTORC1 signaling cascade, the enhanced expression of rps6 would inhibit BTB-related expression in Sertoli cells and in turn impair BTB integrity [36,37].

The assembly and removal of SGs from the male reproductive system appears to be important for male fecundity. One possible regulator is an SG-associated factor, SERBP1, which acts as a regulator of heat-induced SG elimination and regulates 26S proteasome activity and G3BP1 ubiquitination in germ cell survival against HS [38]. Similarly, the important role of SGs in the maintenance of the BTB and Sertoli cell function was confirmed in this study. SGs are important for Sertoli cells since they provide a homeostatic environment resistant to HS for spermatogenesis. This suggests that further exploration of SGs in the male reproductive system is warranted. In addition, because of the critical cellular functions of mRNPs and the possible links to stress-related diseases, the role of SGs in testicular inflammation and disease is also of interest. Whether some testicular diseases and long-term inflammation can induce the formation of SGs and the effect of the clearance and maintenance of these SGs on male fertility is an interesting topic and worthy of further investigation. At present, there are still many gaps and controversies in our understanding of the mechanism of the SG system in male reproduction. And a lot of work remains to be done, such as the expansion of the SG pool within the testis and the demonstration of the role of these markers more fully in vivo.

In summary, our study demonstrates the role of SGs in maintaining BTB functions during HS and identifies UBAP2 as a functional component in heat-induced SGs in Sertoli cells. Our findings not only help to expand our understanding of SGs in male reproduction and the molecular mechanism of low fertility in males in summer but also potentially provide guidance for the development of clinical drugs protecting against HS.

## 4. Materials and Methods

### 4.1. Animals

All the ICR mice aged 8 weeks were purchased from Spf (Sipeifu, Beijing, China) Biotechnology Company. Mice were maintained under standard conditions with free access to food and water in a cycle of 12 h light and 12 h dark. The entire study was reviewed and approved by the China Agricultural University Institutional Animal Care (permission No. AW52503202-1-1) and Use Committee and performed in accordance with the committee’s guidelines. All efforts were made to minimize animal suffering.

### 4.2. Heat Stress of Mice

Before the experiment, 8-week-old male mice were randomly divided into control group and experimental group, with 3 mice in each group. Mice were anesthetized and subjected to HS. The lower 1/3 of the bodies (hind legs, tail, and scrotum) were immersed in a 43 °C water bath for 30 min. For SG detection, testes were removed from the mice immediately and fixed in 4% paraformaldehyde. For testicular seminiferous tubules morphology and BTB integrity, mice were maintained under standard conditions for recovery with different times before testis collection. Standard room conditions were maintained in the non-HS control group.

For CHX treatment, a dosing solution of 10 μg/μL CHX in saline was prepared before treatment. For inhibition of SG formation, mice were injected with different doses of CHX (mentioned in Figure captions) via the tail vein. After half an hour, mice were anesthetized and subjected to HS. Each group contained three mice. Negative controls received PBS injections at room temperature.

### 4.3. Cell Culture

The 293T cells were grown in DMEM (11965092, Gibco, New York, NY, USA) supplemented with 10% (*v*/*v*) fetal bovine serum (C04001, Vivacell, Shanghai, China), 1% (*v*/*v*) MEM NEAA (11140076, Gibco, New York, NY, USA), 1 mM sodium pyruvate (11360070, Gibco, New York, NY, USA), 100 U/mL penicillin and 100 μg/mL streptomycin (15140122, Gibco, New York, NY, USA) in a 37 °C incubator with 5% CO_2_. TM4 cells were grown in DMEM/F12 (11330032, Gibco, New York, NY, USA) supplemented with 10% (*v*/*v*) fetal bovine serum (C04001, Vivacell, Shanghai, China), 100 U/mL penicillin and 100 μg/mL streptomycin (15140122, Gibco, New York, NY, USA) in a 37 °C incubator with 5% CO_2_. Cells were allowed to adhere and to reach 50–70% confluency for HS, siRNA and vector transfection, as described below.

### 4.4. Heat Stress of TM4 Cells

TM4 cells were allowed to adhere and to reach 70% confluency for experiments. Cells were cultured under HS at 43 °C for 30 min, followed by maintenance at 37 °C for 7 h. The above procedure was repeated three times.

For the CHX treatment, cells were replaced with fresh medium containing 20 μg/mL CHX and cultured for 30 min before 43 °C HS for 30 min. Subsequently, cells were replaced with fresh medium to resume culture for 7 h. The above procedure was repeated three times. Cells were collected in liquid nitrogen. Standard conditions with CHX treatment were maintained for the control group.

For the siRNA treatment, after transfection for 48 h, cells were cultured under HS at 43 °C for 30 min, followed by maintenance at 37 °C for 7 h. The above procedure was repeated three times. After the indicated time, the cells were harvested for the subsequent experiments.

For all cell experiments, three biological replicates were assayed.

### 4.5. BTB Permeability Assay

The integrity of BTB was assessed using EZ-Link SulfoNHS-LC-Biotin (21335, Thermo Fisher Scientific, Waltham, MA, USA) as previously described [39]. Biotin was dissolved in PBS containing 1 mM calcium chloride at a concentration of 10 mg/mL. After mice anesthesia and testis HS, the biotin solution was injected into the interstitial area under the tunica albuginea before 30 min incubation (25 μL per testis). Subsequently, the testes were fixed with Bouin’s fixative for subsequent paraffin embedding and section. The sections were stained with FITC-streptavidin (1:200; 4800-30-14, Thermo Fisher Scientific, Waltham, MA, USA) and DAPI (P0131, Beyotime, Shanghai, China). Testicular sections were photographed with a fluorescence microscope. The FITC signal of tracer in the seminiferous tubules was used to indicate BTB damage and imaged using a fluorescence microscope (ScopeA1, ZEISS, Oberkochen, Germany). Three random sections of a whole cross-section from each mouse were examined. The final data were collected from three mice in each group. The proportion of damaged seminiferous tubules to the total seminiferous tubules in the cross-section was used for statistically analysis.

### 4.6. Plasmid Constructs and Transfections

For lentiviral plasmid construction, the cDNA of mouse G3BP1 was cloned into the BsrGI and SaII sites of the pLenti-CMV-GFP plasmid (17448, Addgene, Watertown, MA, USA). HEK293FT cells were seeded on 15 cm plates at approximately 50% confluence and left overnight, and 25 μg of total plasmid (pLenti-CMV-GFP:pSPAX2:pMD2.G = 3:2:1) was co-transfected with Lipofectamine 2000 (11668500, Thermo Fisher Scientific, Waltham, MA, USA) according to the manufacturer’s protocol. Transfected cells were cultured for 48 h under standard conditions and the supernatant was collected. Lentivirus was obtained by centrifugation at 45,000 rpm for 2 h at 4 °C and concentrated using PBS (30 μL per 15 cm dish).

TM4 cells grown to 70% confluency were used for lentivirus transfection. A total of 30 μL of concentrated lentivirus per 3.5cm dish was added. Infected cells were cultured for 48 h under standard conditions. Positive monoclonal G3BP1-GFP TM4 cells were selected using fresh medium containing Puromycin (final concentration 1 μg/mL) and identified by PCR.

Candidate proteins from proteomics were amplified and cloned into the AscI and PacI sites of pDsRed-C1. G3BP1-GFP TM4 cells were seeded on 3.5 cm plates at approximately 50% confluence and left overnight and 2 μg of total plasmid was transfected with Lipofectamine 2000 according to the manufacturer’s protocol. The empty vector was used as control. 48 h after transfection, the transfected cells were subjected to HS (43 °C for 30 min) for colocalization of testis. For the siRNA transfection, all the siRNAs were obtained from Suzhou Genepharma Co., Ltd. (Genepharma, Suzhou, China) and transfected into TM4 using GP-RNA Mate (G04006, Genepharma, Suzhou, China) according to the manufacturer’s instructions. All the primers and siRNA sequences are listed in Supplementary Table S4.

### 4.7. SG Isolation

SG isolation was adapted from previous paper [32,33]. TM4 cells expressing G3BP1-GFP with 80–90% confluency were suitable for experiments. Cell media were replaced with fresh media after 1 h and then treated at 43 °C for 30 min. Standard culture conditions were maintained as the control group (SG-negative). Cells were collected in falcon tube after centrifuging at 1500× *g* for 3 min. The pellets were flash-frozen in liquid N_2_ and placed on ice for 5 min before subsequent isolation.

The pellets were re-suspended in SG lysis buffer (50 mM TrisHCl pH 7.4, 100 mM KOAc, 2 mM MgOAc, 0.5 mM DTT, 50 μg/mL Heparin, 0.5% NP40, protease inhibitor (11836170001, Sigma Aldrich, St Louis, MO, USA), 1 U/μL of recombinant RNase inhibitor (2313A, Takara, Dalian, China)). Syringe with 25 gauge 5/8 needle on ice was used to accelerate lysis. The lysates were centrifuged at 1000× *g* for 5 min at 4 °C to remove cell debris. The supernatant was then spun at 18,000× *g* for 20 min at 4 °C to collect SG cores. SG core pellets re-suspended in 1 mL SG lysis buffer were spun at 18,000× *g* for 20 min at 4 °C again and re-suspended in 300 μL SG lysis buffer. The supernatant was spun at 850× *g* for 2 min at 4 °C. At this stage, the processing supernatant contained the SG-core-enriched fraction.

The SG-enriched fraction was pre-cleared twice by rotating with 30 μL equilibrated dynabeads (10002D, Thermo Fisher Scientific, Waltham, MA, USA) at 4 °C for 30 min. SG-enriched fraction was rotated with 20 μL of anti-GFP antibody at 4 °C for 1 h after removing dynabeads. The SG-enriched fraction was precipitated by spinning at 18,000× *g* for 20 min at 4 °C and re-suspended in 500 μL SG lysis buffer and 60 μL equilibrated Protein A Dynabeads. The mixture was rotated for 3 h at 4 °C. Then, dynabeads were collected and washed using three different wash buffers for 5 min each (wash buffer 1: 20 mM Tris HCl pH 8.0, 200 mM NaCl, 1 U/μL of recombinant RNase inhibitor; wash buffer 2: 20 mM Tris HCl pH 8.0, 500 mM NaCl, 1 U/μL of recombinant RNase inhibitor; wash buffer 3: SG lysis buffer, 2M Urea, 1 U/μL of recombinant RNase inhibitor).

The beads were mixed with 2× loading buffer and incubated at 95 °C for 10 min. Then, these samples were used for proteome sequencing or Western blotting.

### 4.8. Western Blot

Whole cells and tissues were lysed in cell lysis buffer (P0013, Beyotime, Shanghai, China) with a protease and phosphatase inhibitor cocktail (P1045, Beyotime, Shanghai, China) on ice. Centrifugation was performed at 12,000× *g* for 15 min to remove cell debris, followed by quantification using the BCA Protein Assay Kit (P0012, Beyotime, Shanghai, China).

For Western blotting, proteins were separated on sodium dodecyl sulfate polyacrylamide gel electrophoresis gels and electrotransferred to PVDF membranes (1620177, Bio-rad, Hercules, CA, USA). The membranes were incubated with primary antibodies overnight at 4  °C after blocking with 5% skimmed milk in TBST for 1 h. Then, the membranes were incubated with HRP-conjugated secondary antibodies for 1 h at room temperature after washing. The proteins were visualized using Western ECL substrate (1705060, Bio-rad, Hercules, CA, USA) on the Tanon 5200 (5200, Tanon, Shanghai, China) chemiluminescence imaging system. Image analysis software (ImageJ, v1.8.0.345) was used to quantify the band density. The band density of each protein was corrected with β-Actin as the relative expression, which was used for statistical analysis.

Antibodies against GFP (#66002-1-lg), G3BP1 (#66486-1-lg), rpS6 (#66886-1-lg) and phospho-rpS6 (Ser236/236, #29223-1-AP) were from Proteintech (Proteintech, Wuhan, China). Antibodies against Claudin-11 (#HX13363) were from Huaxingbio (Huaxingbio, Beijing, China). Antibodies against TIA-1 (#sc-166247) were from Santa Cruz (Santa Cruz Biotechnology, Shanghai, China). Antibodies against β-Catenin (#BE3365) and FLAG (#BE7003) were from Easybio (Easybio, Beijing, China). Antibodies against ZO-1 (#33-9100) were from Thermo Fisher (Thermo Fisher Scientific, Waltham, MA, USA). Antibodies against β-Actin (#ab49900) were from Abcam (Abcam, Cambridge, Britain). Antibodies against UBAP2 (#PK59381) were from Abmart (Abmart, Shanghai, China).

### 4.9. RT-qPCR

Total RNA was extracted using TRIzol Reagent (T9424, Sigma Aldrich, St Louis, MO, USA) and reverse transcribed into cDNA using Fastking RT kit (KR116-02, Tiangen, Beijing, China) according to the manufacturer’s instructions. The specific quantitative primers are listed in Supplementary Table S4. A set of cDNA, 2× SuperReal PreMix Plus SYBR Green (FP205, Tiangen, Beijing, China) and forward and reverse primers were mixed and subjected to RT-qPCR performed on CFX96 Touch Deep Well Real-Time PCR Detection System (CFX96, Bio-rad, Hercules, CA, USA). The program was as follows: 95 °C for 15 min, followed by 40 cycles of 95 °C for 10 s, 60 °C for 20 s and 72 °C for 30 s. The reaction was performed in a CFX96™ Real-Time PCR machine (CFX96, Bio-rad, Hercules, CA, USA). The standard curve was used to determine PCR efficiency. The dissociation curve of amplified products was used to evaluate product specificity. Relative expression levels were normalized to β-Actin using the 2^−ΔΔCt^ method. For each gene, three technical replicates and three biological replicates were assayed.

### 4.10. Immunofluorescence

Testes were fixed in 4% paraformaldehyde at 4 °C for 48 h. The fixed samples were embedded in paraffin and cut into 5 μm sections for immunostaining after being dewaxed and rehydrated. After subjecting to antigen retrieval with 0.01 M sodium citrate buffer, testicular sections were blocked with 5% normal goat serum at room temperature for 1 h. Then, sections were incubated with primary antibodies at 4 °C overnight, followed by PBST washing and secondary antibody incubation. Before microscopic imaging, slides were mounted with antifade mounting medium with DAPI (H1200, Vector Laboratories, Burlingame, CA, USA). Cells grown in slides were fixed in 4% paraformaldehyde for 15 min and permeabilized with 0.5% Triton X-100 in PBS for 20 min. The process of blocking and antibody incubation was consistent with the tissue section. The signal was imaged using a fluorescence microscope (ScopeA1, ZEISS, Oberkochen, Germany).

Antibodies against G3BP1 (#66486-1-lg) were from Proteintech (Proteintech, Wuhan, China). Antibodies against β-Catenin (#BE3365) were from Easybio (Easybio, Beijing, China). Antibodies against Occludin (#33-1500) were from Thermo Fisher (Thermo Fisher Scientific, Waltham, MA, USA). Antibodies against SOX9 (T55400) were from Abmart (Abmart, Shanghai, China). Antibodies against Vimentin (#5741S) were from Cell Signaling Technology(Cell Signaling Technology, Danvers, MA, USA).

### 4.11. Statistical Analysis

Experiments were independently replicated three times, and all data were reported as the mean ± SEM. *T*-test was used to analyze the significant difference between the treatment and control groups (two groups). Multiple comparison was assessed using one-way ANOVA with Dunnett’s post hoc test using SPSS 24.0 software. For all statistical analyses, * = *p* ≤ 0.05, ** = *p* ≤ 0.01, *** = *p* ≤ 0.001, n.s. = not significant.

## Data Availability

The datasets for this study are publicly available.

## Supplementary Materials

The following supporting information can be downloaded at https://www.mdpi.com/article/10.3390/ijms25073637/s1.
