# Supplementary material for: The Role of Heat-Induced Stress Granules in the Blood–Testis Barrier of Mice
Source: Int J Mol Sci. 2024 Mar 25;25(7):3637. doi: 10.3390/ijms25073637 (PMC11011666; doi:10.3390/ijms25073637)
Supplement: Supplementary file 1 [file ijms-25-03637-s001.zip › Figure S1-S3.pdf]

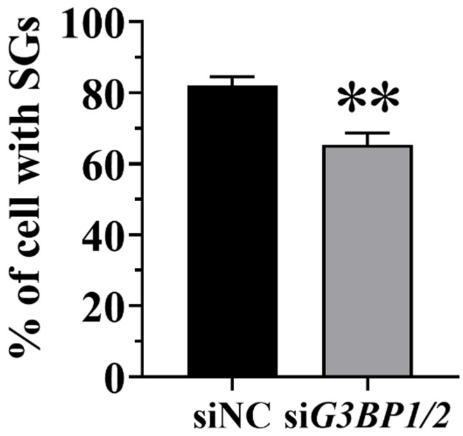

Supplementary Figure S1. Quantitative analysis of si*G3bp1*/*2* on SG assembly in TM4 cells during HS. Cells were transfected with siNC or si*G3bp1*/*2* for 48 h followed by HS at 43℃ for 30 min.

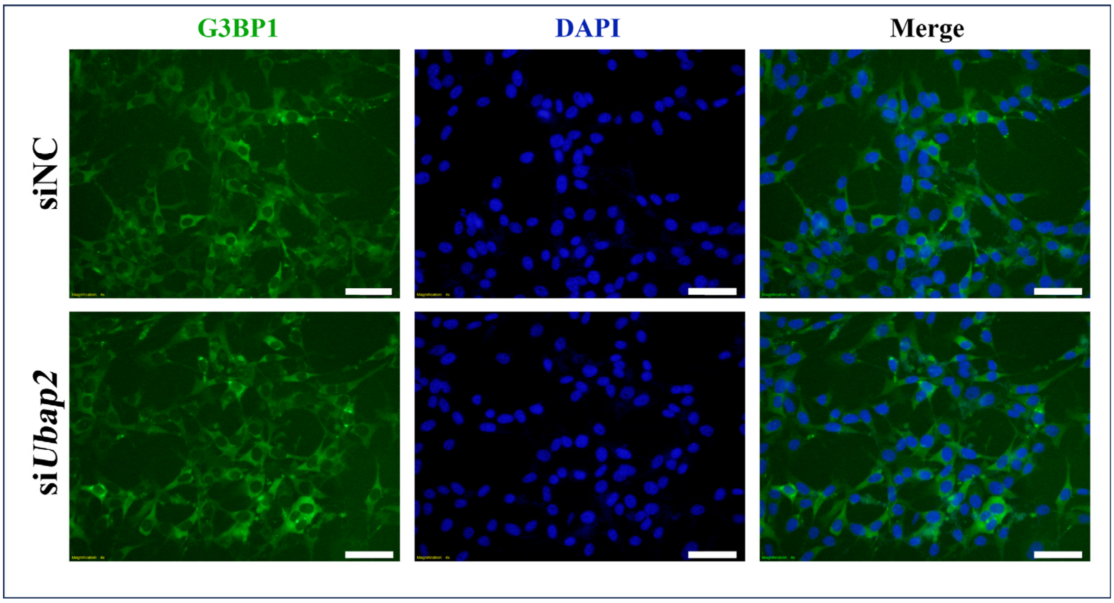

Supplementary Figure S2. Effect of si*Ubap2* on SG disassembly in TM4 cells after HS. Cells were transfected with siNC or si*Ubap2* for 48 h followed by HS at 43°C for 30 min and recovery at 37°C for 90 min. Scale bars, 25 μm.

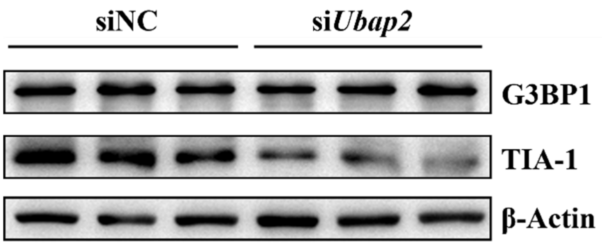

Supplementary Figure S3. Immunoblots of G3BP1 and TIA-1 in non-HS condition.
Cells were transfected with siNC or si*Ubap2* for 48 h at 37°C followed by proteins extraction.
